# Supplementary material for: Comparisons of genome assembly tools for characterization of Mycobacterium tuberculosis genomes using hybrid sequencing technologies
Source: PeerJ. 2024 Aug 29;12:e17964. doi: 10.7717/peerj.17964 (PMC11366230; doi:10.7717/peerj.17964)
Supplement: Supplemental Information 2 [file peerj-12-17964-s002.docx]

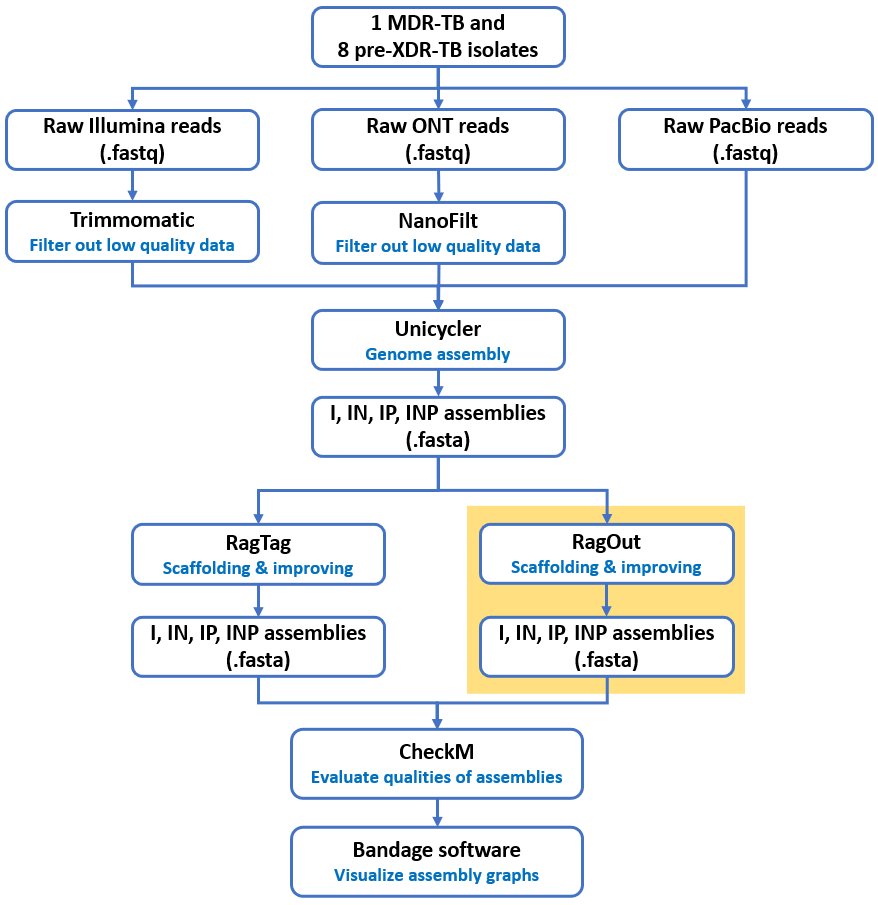
**Supplemental** **Fig. S1** An analysis workflow to generate genome assembly of nine DR-TB isolates using Unicycler, RagOut, and RagTag.

**Supplemental**
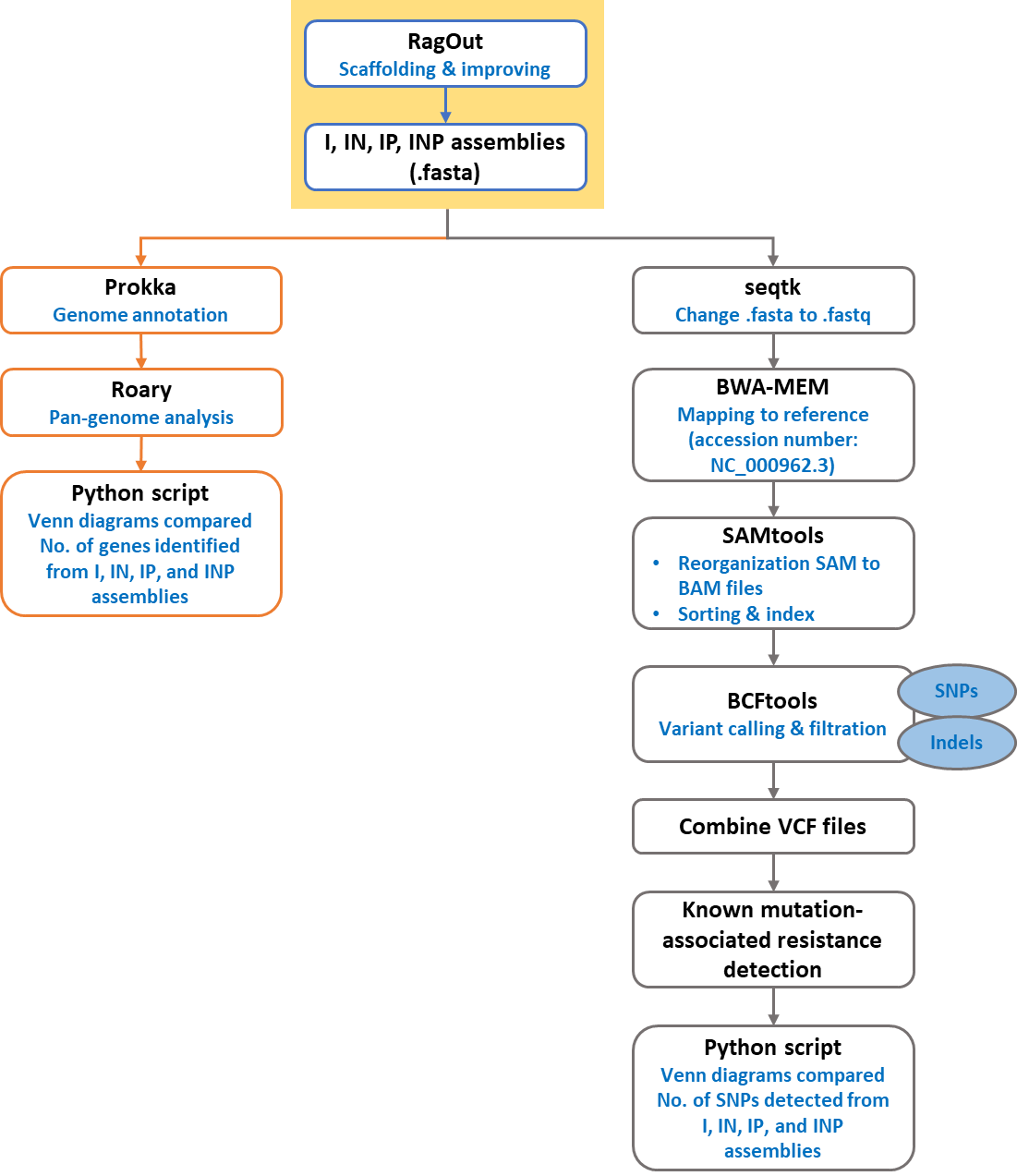
**Fig. S2** An analysis workflow for pan-genome analysis and mutation-associated drug resistance detection.
